# The unexpected evolution of myocardial injury while infected with the coronavirus: A COVID-19 case report

**DOI:** 10.17159/2078-516X/2022/v34i1a11110

**Published:** 2022-01-01

**Authors:** J T Doran

**Affiliations:** 1 Jean Doran Biokineticist, Letterkenny, Derry and Strabane, Ireland and United Kingdom; 2 Director at Just Kinetics Preventive Health Care Provider, Ireland, United Kingdom and South Africa

**Keywords:** electrocardiography, long-haul COVID, pericarditis, gastritis

## Abstract

**Background:**

A novel virus breakout in December 2019, with diverse clinical manifestations, initially identified as infecting the respiratory system, has spread rapidly around the world, with adverse effects which have caused acute myocardial injury and chronic damage to the cardiovascular system in some individuals.

**Aim:**

To present a clinical case with the manifestation of COVID-19 suspected to be either a mild case of either myocarditis or pericarditis. This case highlights a relatively atypical presentation of COVID-19 and the value of a coordinated approach to the unexpected sequences of patient recovery patterns that may require further specialist referral and intervention.

**Findings:**

A ribonucleic acid (RNA) viral infection was confirmed by a polymerase chain reaction with reverse transcription (RT-PCR) and the patient was diagnosed with coronavirus disease 2019 (COVID-19). The presenting symptoms failed to resolve and the patient was admitted to the accident and emergency (A&E) department. Upon the second visit to the A&E department at 27 days postinfection, an electrocardiograph (ECG) was conducted revealing T wave inversion.

**Implications:**

A coordinated approach is needed to combat the infection, develop cardiac-protective strategies and direct supportive measures.

## Case report

Ten months into the pandemic, a fifty-year-old female, having received a positive RT-PCR result, continued to follow public health advice and health authority protocols to self-isolate and stay at home. At the time of the infection, the patient experienced mild symptoms such as a sore throat, headache and what felt like a postnasal drip. The subsequent worsening of the symptoms and the onset of tachycardia on Day 13 postinfection caused her to become extremely anxious and resulted in her attending the local emergency department at Letterkenny University Hospital.

Although the patient had a history of hypertension and osteoarthritis for which she took no medication, she undertook activities of daily living, but did not partake in regular or structured physical exercise. The sudden onset of pleuritic back pain behind the right shoulder blade which persisted centrally, a persistent cough, chest pain, indigestion, severe shortness of breath and an elevated resting heart rate varying between 96 – 115 bpm resulted in the patient not only attending but also being admitted to the A&E department of the local general hospital, Letterkenny University Hospital in the Republic of Ireland. The patient also self-reported the following symptoms: some relief brought on by her administering paracetamol, the back pain was not exacerbated by movement or by deep inspiration, a feeling of her heart racing (observing a resting heart rate of 121 bpm on her Fitbit), two nights of indigestion and pain, whilst sleeping sitting up in bed. On initial assessment, the vital signs indicated no immediate emergency distress, with her BP (blood pressure) 136/89 mmHg, temperature 36°C, pulse 103 bpm (beats per minute), RR (respiratory rate) 20 bpm (breaths per minute) and SAO2 (Saturated Oxygen) 95%. The ECG conducted indicated T-wave inversion, after which she was assigned a yellow triage status. The patient was then discharged and referred to cardiology for cardiac investigations without receiving further treatment. It is unclear if the patient was assessed as having pre-existing T wave changes during this assessment.

On Day 27, the patient was once again admitted to A&E department as the prior symptoms failed to resolve themselves. A more thorough assessment of her medical history revealed the occurrence of sudden cardiac death in the family (father (53), paternal grandfather (54) and a paternal male cousin in his early (50s)). At this time the patient was self-administering ARCOXIA, a non-steroidal anti-inflammatory (NSAID) for knee pain. A reviewed assessment of the vital signs revealed slightly higher levels of all the vitals evaluated when compared to the initial visit on Day 13, with BP 145/88 mmHg, temperature 36.5°C, pulse 108 bpm, respiratory rate 20 breaths per minute and SAO_2_ 97%. The subsequent ECG showed T wave inversion in leads V2, V3, V4, confirming the initial ECG findings obtained at the previous admittance and assessment on Day 13. The patient was now assigned to an orange triage category and kept for further monitoring and investigation. The full blood count (FBC) and differential were normal, with essentially normal liver function, normal renal function, with a glomerular filtration rate (GFR) > 90 ml/min/1.73 m^2^ and creatinine 1.01 mg/dl. However, the fasting lipogram results that were obtained were as follows: total cholesterol 7.4 mmol/l, triglycerides 3.9 mmol/l, high-density lipoprotein (HDL) 1.35 mmol/l, low-density lipoprotein (LDL) 5.6 mmol/l. The above normal levels were of reasonable concern as these may be associated with an increased risk of atherosclerosis and therefore there is the possibility that the patient could be suffering a heart attack. Ongoing inpatient monitoring over the next two weeks revealed that the patient reported palpitations and experienced episodes of intermittent tachycardia, which was revealed to be a normal sinus rhythm according to the ECG tracings. She also had a normal troponin (TnI) level < 0.03 ng/ml and a normal chest X-ray (CXR). The patient was then discharged from the A&E department on Day 44, without undergoing any further treatment for a diagnosis of epigastric pain of unknown origin. Subsequently, it was arranged for her to wear a Holter for five days to monitor any cardiac arrhythmias. She was also referred for an oesophagogastroduodenoscopy (OGDS) to exclude any GIT causes. She was advised to lose weight and to stop taking the ARCOXIA. Medications that were prescribed on discharge to help manage her symptoms included proton pump inhibitors. On Day 50, an outpatient follow-up ECG was conducted and revealed a normal sinus rhythm with a right axis deviation. This was interpreted as a possible right ventricular hypertrophy and evidence of possible prior lateral infarct (see Fig.1b).

On Day 57, following the administration of the Astra Zeneca COVID-19 vaccine the patient experienced extreme palpations, as well as breathlessness and fatigue. On Day 58 further cardiac investigations were carried out as an outpatient and confirmed previous ECG findings, showing a normal sinus rhythm and T wave inversion in leads V2, V3, V4. An echocardiogram showed normal left ventricle size and function with no significant valve disease. The exercise stress test was terminated prematurely after four minutes as her heart rate rapidly accelerated to 160 bpm, with concomitant chest pain and very minor ST changes which resolved quickly during recovery. Also on Day 58, a computed tomography pulmonary angiogram (CTPA) of the lungs showed atelectasis of the lower lungs. There was also the presence of minor atherosclerotic plaque in the left anterior descending artery (LAD), which was not considered significant. A resultant recommendation was made to consider non-atherosclerotic causes of chest pain, as well as the ischemic cardiac changes observed on the ECG. Additional advice included considering preventive therapy and risk modification for coronary artery disease.

On Day 77 the patient presented to a cardiac clinic in South Africa with palpitations and tachycardia. On examination, her blood pressure was measured at 140/80 mmHg with a resting pulse rate of 90 bpm, a respiration rate of 12 breaths per minute with clear air entry bilaterally, and no evidence of structural lung disease. During this review, the medical history was consistent with previous reports, including a list of medications indicating alternative NSAIDs for knee pain other than ARCOXIA as advised. The resting ECG showed a sinus tachycardia of 110 bpm with noted ST/T wave depression from V4 – V6. This was also noted inferiorly and at this stage, it was unclear if the ST/T wave depression was old or new. In addition, a QRS duration of 80 ms and a QRS axis of approximately 70°+ was noted. A subsequent treadmill test determined that a maximum heart rate was rapidly achieved. The total exercise time was 2 minutes and 12 seconds with a maximum heart rate of 160 bpm equating to 94% of maximum heart rate and a target of 4.17 METs. No supraventricular tachycardia or inducible ventricular tachycardia was documented. The patient was generally deconditioned and in addition had mobility limitations due to her right knee problem. A repeat echocardiogram determined the aortic, pulmonary, tricuspid and mitral valves were all within normal limits and without defect or abnormalities. The patient’s presenting clinical features were reported to be not in keeping with postural orthostatic tachycardia syndrome (POTS). Autoimmune dysfunction was not excluded. It was also suspected that she may be suffering from a mild form of myo-and/or pericarditis. Recommendations were made to follow conservative therapy and lifestyle modifications. Depending on the response to this therapy, further electrophysiological opinion should be considered and which may also include a Holter assessment. Further investigations to be considered included a gastroscopy. There is no record of a creatine kinase (CK), a C-reactive protein (CRP) or a D-dimer being carried out.

Therefore the complete but rather broad differential diagnosis included: possible long-haul COVID, mild myocarditis, pericarditis, pleuritis and or gastritis.

### Treatment

The patient subsequently sought supportive measures and self-medicated with Neem powder which she stated helped to alleviate the shortness of breath and fatigue after/within four days. The use of Ulsanic syrup (Sucralfate) greatly relieved the chest and gastric distress suggesting a possible gastric contribution to her symptoms. The patient has subsequently followed the recommendations of conservative therapy and lifestyle modifications and has received a dietary prescription from a dietician. She also consulted a biokineticist for cardiac rehabilitation and exercise therapy. The patient seems to have recovered well and is fully functional. She has returned to work, started a new job, and taken on more responsibility.

## Discussion

### Virology

It is currently thought that COVID-19 infection can be associated with myocardial damage.^[1]^ The pathogenesis of COVID-19 disease may include invasion of several essential organs which may result in multiple organ failure and a hyperimmune response, such as the now well-described cytokine storms.^[1–4]^ The heart is one of the potentially most critically affected organs and the infection may contribute to myocardial damage. Other viral illnesses, such as influenza, have also been associated with myocardial inflammation such as the development of myocarditis as reported by Anupama et al.^[5]^

### Pathophysiology

The pathogenesis of myocardial injury in affected patients postinfection remains unclear.^[4, 6–7]^ The current hypothesis is that the pathogen binds itself to a functional receptor and gains entry into the cell using the angiotensin-converting enzyme 2 (ACE2) to accomplish this.^[8]^ Patients with prevalent cardiovascular disease and comorbidities seem to be more significantly associated with morbidity.^[3]^ While it is considered that COVID-19 causes viral pneumonia with extrapulmonary manifestations, additional possible major complications are thought to include acute myocardial injury, acute myopericarditis, arrhythmia, shock and long-term chronic damage. Related studies, such as the study by Guistino^[4]^, suggest that infection may result in cardiac injury via the following proposed mechanisms: cytokine-mediated damage, oxygen supply/demand imbalance, ischemic injury from microvascular thrombi formation and direct invasion by the pathogen of the myocardium. Gastroesophageal reflux which is uncontrolled acid reflux or ingestion may also contribute to the risk of developing interstitial pneumonia. In this case, the pathophysiology and mechanism of cardiac injury remains uncertain.

### Electrocardiography

The use of electrocardiography may be useful in the early diagnosis of myocardial injury; however, limited details are available on the changes in electrocardiography linked to myocardial injury post-infection. There seems to be consistent evidence of sinus tachycardia and ST depression in the anteroseptal leads irrespective of disease severity.^[1]^ ECG changes as observed in this case can be associated with mild infection and may only present with myocardial necrosis markers in 2–4% of patients, according to Li et al.^[3]^ The ECG investigation at 77 days post-infection reports an elevated heart rate of 110 bpm, a QRS axis of approximately 70°+ which is a notable change from the ECG on Day 50 (Fig. 1b) and which may suggest some clinical improvement in reversible electrocardiographic changes or resolving of the conduction disturbance. There seems to be more prominent T wave changes when comparing the first ECG (Fig. 1a) to the second (Fig. 1b), most evident in lead V4, with some evidence to suggest the presence of a T wave inversion pre-infection in lead V3. The T wave abnormalities that were noted in the anterior leads may be the only evidence of mild myocarditis as all cardiac bloods were normal and the patient had a resting tachycardia. Leads 1 and AVL appear quite different in the second ECG (Fig. 1b) when compared to the first ECG (Fig. 1a), suggesting a possible unexplained high lateral conduction disturbance; similarly AVR is also altered in comparison. The persistence of sinus tachycardia could be suggestive of many things including the presence of cardiac inflammation and that in some way the heart is still being influenced.

## Conclusion

There is limited understanding of the transmission dynamics and spectrum of clinical illness of COVID-19. Cardiac involvement with various ECG presentations is possible and clinicians should be aware of this possibility. Pericardial and myocardial inflammation may prompt symptoms, yet may precede the generation of an observable pericardial effusion. Early management and treatment of the causative disease is critical to prevent the spread of the pathogen and to improve the cure/recovery rates. With particular focus on trying to prevent progression to the inflammatory phase of the disease (i.e. cytokine storm). This case highlights an atypical presentation of COVID-19 infection with cardiac involvement and non-specific ECG changes that may present pericarditis and/or mild myocarditis or a subtle ischaemic event. Healthcare teams need to consider a coordinated approach for these patients to ensure the correct diagnoses of possible sequelae are made timeously and the appropriate management applied. Just as important the clinical management in the acute phase is vital, and so is the management of the recovery phase with the emerging understanding of the role various pharmaco- and physical therapy may have to play. This patient had various risk factors known to be associated with worsening morbidity following COVID-19 infection, including hypertension and relative physical inactivity. A comorbidity of hypertension, physical inactivity, poor lifestyle and ageing also associated with a higher risk for severe COVID-19 outcomes. Therefore improving lifestyle factors such as physical activity status (with better BMI, blood pressure control, etc) would presumably be of benefit not only for the patient’s recovery but also in the reduction of the risk of severe disease for future re-infections with the virus. Cardiac-protective strategies pre-infection have been shown to include key contributing factors, such as a healthy immune system, mitochondrial health and consistent physical activity that meets physiologically enhancing levels.

It is of interest to note that this patient utilised Neem powder, a naturopathic medication which she felt helped reduce her symptoms. Neem powder, which is extracted from Neem leaves as a natural medicine, was administered by the patient as an alternative to allopathic medicine. Neem leaves, purported to have antiviral and anti-inflammatory properties, might be used by some as a treatment against viral infection and as a possible prophylaxis pre-infection. But further research is needed to determine if there is a role for it in the management and prevention of COVID-19.

This case report may help in the future treatment of patients with this unique clinical presentation. Further clinical studies are needed to evaluate the exact role of the cardiac-protective strategies as suggested in this case, this patient remains part of an ongoing investigation.

## Figures and Tables

**Fig. 1a:**
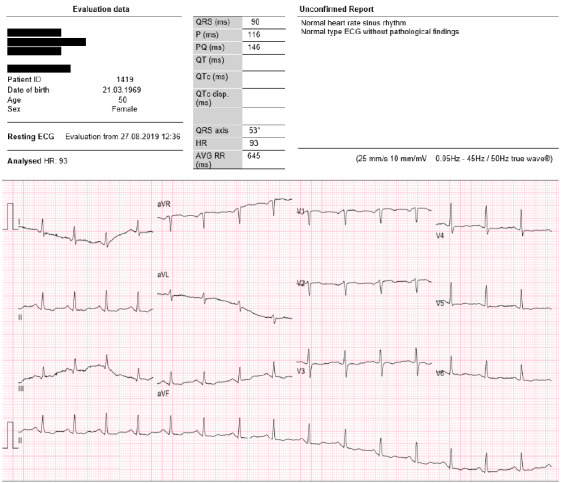
ECG of the heart (18 months prior to infection)

**Fig. 1b:**
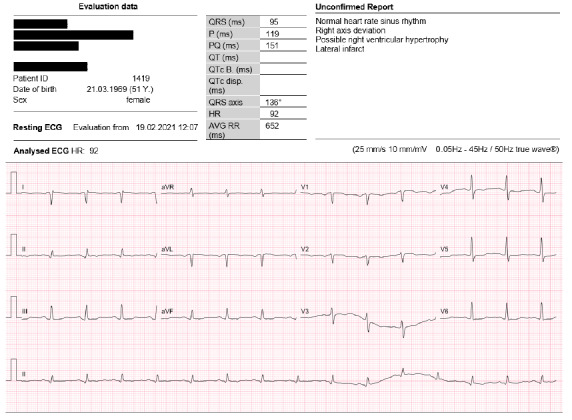
ECG of the heart (Day 50)
